# Circulating microRNAs as a Fingerprint for Liver Cirrhosis

**DOI:** 10.1371/journal.pone.0066577

**Published:** 2013-06-21

**Authors:** Yan-Jie Chen, Ji-Min Zhu, Hao Wu, Jia Fan, Jian Zhou, Jie Hu, Qian Yu, Tao-Tao Liu, Lei Yang, Chun-Lei Wu, Xiao-Ling Guo, Xiao-Wu Huang, Xi-Zhong Shen

**Affiliations:** 1 Department of Gastroenterology, Zhongshan Hospital of Fudan University, Shanghai, China; 2 Liver Cancer Institute, Zhongshan Hospital of Fudan University, Shanghai, China; 3 Department of Statistics, School of Public Health of Fudan University, Shanghai, China; 4 Department of Molecular and Experimental Medicine, The Scripps Research Institute, La Jolla, California, United States of America; Northwestern University Feinberg School of Medicine, United States of America

## Abstract

**Background:**

Sensitive and specific detection of liver cirrhosis is an urgent need for optimal individualized management of disease activity. Substantial studies have identified circulation miRNAs as biomarkers for diverse diseases including chronic liver diseases. In this study, we investigated the plasma miRNA signature to serve as a potential diagnostic biomarker for silent liver cirrhosis.

**Methods:**

A genome-wide miRNA microarray was first performed in 80 plasma specimens. Six candidate miRNAs were selected and then trained in CHB-related cirrhosis and controls by qPCR. A classifier, miR-106b and miR-181b, was validated finally in two independent cohorts including CHB-related silent cirrhosis and controls, as well as non−CHB-related cirrhosis and controls as validation sets, respectively.

**Results:**

A profile of 2 miRNAs (miR-106b and miR-181b) was identified as liver cirrhosis biomarkers irrespective of etiology. The classifier constructed by the two miRNAs provided a high diagnostic accuracy for cirrhosis (AUC = 0.882 for CHB-related cirrhosis in the training set, 0.774 for CHB-related silent cirrhosis in one validation set, and 0.915 for non−CHB-related cirrhosis in another validation set).

**Conclusion:**

Our study demonstrated that the combined detection of miR-106b and miR-181b has a considerable clinical value to diagnose patients with liver cirrhosis, especially those at early stage.

## Introduction

Liver cirrhosis, one of the most common non-neoplastic causes of mortality worldwide, is defined as the histological development of regenerative nodules surrounded by fibrous connective tissue in response to chronic liver disease [1]. Though the exact prevalence of cirrhosis is unknown, it was estimated at 800,000 deaths each year worldwide [2].

Recently, with great advancements in treating chronic liver diseases, the accurate assessment of silent (i.e., clinically asymptomatic) cirrhosis has become an urgent need for optimal individualized management of disease activity in patients [3]–[5]. However, the lack of accurate, reproducible and easily applied methods for silent cirrhosis has been the major limitation for both clinical management and research purposes. Liver biopsy, the golden standard for fibrosis assessment, still has some limitations including morbidity and mortality, observer variability, and sampling variation [6]. Therefore, developing a reliable noninvasive and convenient method for evaluating histological changes will be a major advance in diagnosis of liver cirrhosis. Currently the noninvasive diagnosis of liver cirrhosis mainly relies on the combination of imaging and serological markers, but none of them are completely satisfactory [7]. Thus the identification of accurate and validated predictive and prognostic markers for liver cirrhosis is needed to improve diagnosis, guide molecularly targeted therapy and monitor activity and therapeutic response.

MicroRNAs (miRNAs) are a class of evolutionarily conserved small (18–24 nucleotides) non-coding RNAs that regulate expression of genes at post-transcriptional level [8], [9]. They have been shown to be involved in a number of critical biological processes, including cell development, differentiation and apoptosis [10]. In addition, substantial studies have also identified miRNAs in circulation to serve as biomarkers for diverse diseases including chronic liver diseases [11], [12]. To explore the clinical applicability of miRNAs as non-invasive circulating biomarker for the assessment of liver cirrhosis, a retrospective case-control analysis was conducted utilizing four independent cohorts including 398 participants. A genome-wide miRNA microarray was first performed in 80 plasma samples. Six candidate miRNAs were selected and then trained in chronic hepatitis B (CHB)-related cirrhosis and controls as primary training sets, and two of them in advanced training sets. A classifier, miR-106b and miR-181b, was validated finally in two independent cohort including CHB-related silent cirrhosis (liver cirrhosis with no biochemical or clinical manifestations irrespectively of etiology) and controls, as well as non−CHB-related silent cirrhosis and controls as validation sets, respectively. Our studies provided promising, albeit preliminary evidence, that combination of miR-106b and miR-181b correlates with histological features of progression and harbors the potential to emerge as a diagnostic biomarkers for all types of liver cirrhosis.

## Methods

### Study Design and Participants

In this study, we included 128 CHB-related cirrhosis patients, 79 CHB patients, 47 non−CHB-related cirrhosis patients, 7 non−CHB-related chronic liver disease patients, and 137 healthy individuals. Healthy individuals were identified by absence of liver symptoms, history of liver disease, and normal liver function. Patient selection criteria were defined according to the American Association for the Study of Liver Diseases Practice Guidelines (Table S1 in File S1). Patients with any acute disease exacerbation, history of cancer, and any active medical condition, such as diabetes, immunosuppression, or coronary artery disease, were excluded from the study. The protocol was approved by the ethics committee (ethics committee of Zhongshan Hospital of Fudan University, Shanghai), and written informed consent was obtained from all study participants.

**Figure 1:**
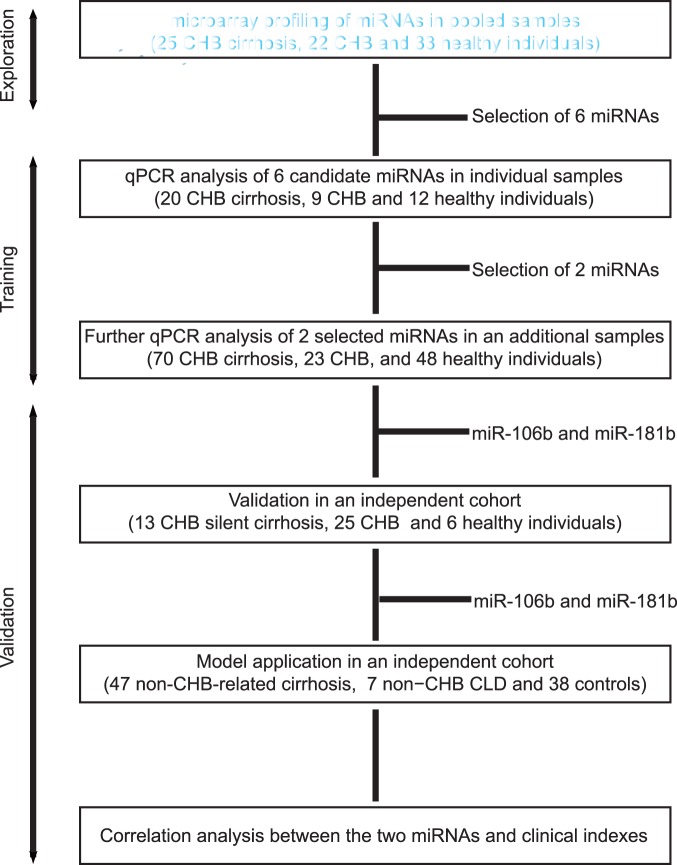
Overview of the design strategy. This study was divided into three phases: exploration, training and validation phase. CHB, chronic hepatitis B; CHB cirrhosis, CHB-related cirrhosis; non−CHB CLD, non−CHB-related chronic liver disease; qPCR, quantitative real-time PCR.

A multistage, case-control study was designed to evaluate plasma miRNAs as candidate biomarkers in patients with liver cirrhosis (Fig. 1). In the primary exploration phase, eighty plasma samples, each with 723 microRNAs, were screened using human miRNA microarrays based on the data described in our recently published study [13]. Of the 80 samples, 25 had CHB-related cirrhosis and 22 had CHB, together with the remaining 33 as healthy controls. A biomarker confirmation analysis was subsequently performed with qPCR assay to refine the number of serum miRNAs in the liver cirrhosis. This analysis was carried out in 2 phases: (a) the training phase, in which plasma samples from 20 CHB-related patients, 9 CHB patients and 12 healthy controls were tested for initial evaluation of six candidates discovered; then 70 CHB-related cirrhosis, 23 CHB, and 48 healthy individuals were tested for evaluation of the two selected miRNAs (Fig. S1 in File S1), and (b) the validation phase, in which plasma samples from 13 CHB-related silent cirrhosis, 25 CHB, and 6 healthy controls (all of them had liver biopsy) were tested for definitive evaluation; as well as 47 non-CHB-related cirrhosis, 7 non−CHB-related chronic liver disease and 38 healthy individuals were finally tested for model application (Table 1).

**Table 1:** Characteristics of the whole participants.

Characteristics of Study Subjects on Microarrays											
				CHB cirrhosis			CHB			Healthy	
(n)				25			22			33	
Age (years)				49 (38, 60)			42 (31, 53)			43 (29, 57)	
Gender		Male		19 (76%)			18 (82%)			13 (39%)	
		Female		6 (24%)			4 (18%)			20 (61%)	
**Characteristics of Study Subjects on qPCR:**											
				**CHB cirrhosis**	**Non-CHB cirrhosis**			**CHB**	**Non-CHB chronic liver disease**		**Healthy**
**(n)**				103	47			57	7		104
**Age (years)**				48.9 (20, 80)	59.2 (39, 80)			36.5 (19, 62)	40 (27, 62)		47.95 (21, 69)
**Gender**			**Male**	84 (81.6%)	27 (57.4%)			41 (71.9%)	4 (57.1%)		76 (73.1%)
			**Female**	19 (18.4%)	20 (42.6%)			16 (28.1%)	3 (42.9%)		28 (26.9%)
**TBIL (µmol/L)**			**<20.4**	83 (80.6%)	30 (63.8%)			47 (82.5%)	6 (85.7%)		103 (99%)
			**>20.4**	20 (19.4%)	17 (36.2%)			10 (17.5%)	1 (14.3%)		1 (1%)
**ALT (U/L)**			**<75**	83 (80.6%)	39 (83.0%)			35 (61.4%)	2 (28.6%)		102 (98%)
			**>75**	20 (19.4%)	8 (17.0%)			22 (38.6%)	5 (71.4%)		2 (2%)
**E antigen**			**Positive**	33 (32.0%)	–			31 (54.4%)	–		–
			**Negative**	70 (68.0%)	–			26 (45.6%)	–		–
**ALB (g/L)**			**<35**	50 (48.5%)	33 (70.2%)			5 (8.8%)	2 (28.6%)		0 (0%)
			**>35**	53 (51.5%)	14 (29.8%)			52 (91.2%)	5 (71.4%)		104 (100%)
**PT (s)**			**<13**	17 (16.5%)	13 (27.7%)			49 (86.0%)	5 (71.4%)		–
			**>13**	86 (83.5%)	34 (72.3%)			8 (14.0%)	2 (28.6%)		–
**INR**			**<1.2**	45 (43.7%)	22 (46.8%)			55 (96.0%)	7 (100%)		–
			**>1.2**	58 (56.3%)	25 (53.2%)			2 (4.0%)	0 (0%)		–
**Ascites**				45 (43.7%)	25 (53.2%)			0 (0%)	0 (0%)		0 (0%)
**Hepatic encephalopathy**				3 (2.9%)	1 (2.1%)			0 (0%)	0 (0%)		0 (0%)
**Variceal hemorrhage**				60 (58.3%)	28 (59.6%)			0 (0%)	0 (0%)		0 (0%)
**Child Pugh**	**A**			63 (61.2%)	22 (46.8%)			–	–		–
	**B**			29 (28.2%)	22 (46.8%)			–	–		–
	**C**			11 (10.6%)	3 (6.4%)			–	–		–
**A detailed classification of non−CHB cirrhosis (n (%))**											
**Alcoholic cirrhosis**				**Schistosomiasis cirrhosis**		**Autoimmune cirrhosis**			**Biliary cirrhosis**		**Others**
16 (34.0%)				9 (19.1%)		9 (19.1%)			4 (8.5%)		9 (19.1%)

CHB, chronic hepatitis B; CHB cirrhosis, CHB-related cirrhosis; qPCR, quantitative real-time PCR; TBIL, total bilirubin; ALT, alanine aminotransferase; PT, prothrombin time; INR, international normalized ratio.

### Sample Collection and miRNA Isolation

To obtain plasma, the blood (2 ml) was obtained on admission and transferred into tubes containing EDTA as the anticoagulant. The blood was then centrifuged (820 g for 10 min at 4°C) to obtain plasma. Plasma samples were stored at –80°C until analysis. Total RNA from the plasma samples was isolated simultaneously with the miRcute miRNA Isolation Kit from Tiangen (Beijing, China) according to the manufacturer’s protocol.

### Microarray and Data Analysis

Total RNA labeled with Cy3 was extracted by using mirVana PARIS miRNA isolation kit according to the instruction from Ambion (Austin, TX, USA), and the concentration was quantified by NanoDrop 1000 Spectrophotometer from NanoDrop Technologies (Waltham, MA, USA). Human miRNA microarrays from Agilent Technologies (Santa Clara, CA, USA) containing probes for 723 human miRNAs were used in plasma samples. Each slide is formatted with eight identical arrays. Total RNA (100 ng) derived from plasma samples were labeled with Cy3. XDR Scan (PMT100, PMT5) was used to scan microarray slides. The labeling and hybridization were performed according to the protocols in the Agilent miRNA microarray system. Feature Extraction Software Rev. 9.5.3 from Agilent Technologies (Santa Clara, CA, USA) was used to convert the microarray image information into spot intensity values. The signal after background subtraction was directly exported into the GeneSring GX10 software from Agilent Technologies (Santa Clara, CA, USA). The raw signals of single color CY3 hybridization were normalized by a stable endogenous control miR-1228 and transformed to log-2 scale. All assays were carried out in triplicate. A sample that showed intra-array coefficients of variation (CV) across replicated spots on an array above 15% or positive signals less than 5% was considered to be unreliable and excluded from further analysis. The miRNA, which had positive signals on microarrays in more than 50% of plasma samples from any one of the subjects, was chosen as a detectable miRNA.

### Quantitative Real-time PCR (qPCR) of miRNAs

Uniform procedures, reagents, and equipment were used to ensure consistency in experimental conditions. Staff involved in experimental procedures was jointly trained. All reagents, including all qPCR reagents, were ordered in one batch, centrally stored. Furthermore, all the qPCR reactions were performed on the same equipment. As to internal reference, expression of miR-16 or miR-1228 was homogeneous in all 80 samples for the microarray; however amplification significantly affects the stability of miR-1228, thus miR-16 served as internal reference in qPCR.

One hundred and forty nanogram plasma miRNA was polyadenylated by poly (A) polymerase in a 20 µL volume, and 6 µL of the poly (A) reaction solution was reverse transcribed to cDNA in another 20 µL using miRcute miRNA First-strand cDNA Synthesis Kit from Tiangen (Beijing, China) following the manufacturer’s protocol. The reaction solution including cDNA products was diluted to one tenth with RNase free water and stored at −20°C for future use. The PCR reaction was performed using miRcute miRNA qPCR Detection Kit from Tiangen (Beijing, China) in Mastercycler® ep realplex from Eppendorf (Hamburg, Germany). Each qPCR contained 9 µL of diluted cDNA, 10 µL of 2× miRcute miRNA premix (with SYBR and ROX), 200 nM manufacturer provided universal reverse primer, and 200 nM miRNA-specific forward primer from Sangon Biotech (Shanghai, China) (Table S2 in File S1). Each sample in duplicates was assessed in 45 cycles of denaturation for 20 s at 94°C, annealing for 30 s at 60°C, and extension for 32 s at 72°C. At the end of the PCR cycles, melting curve analysis were performed to validate the specificity of the expected PCR product. The relative expression of each miRNA was calculated from the equation 2^−Δ^CT with miR-16 as the endogenous control to normalize the data. ΔCT was calculated by subtracting the CT values of miR-16 from the CT values of the miRNAs of interest [14].

### Statistical Analysis

Data were analyzed using either R (version 2.15.1) or SPSS (version 19.0). For microarray analysis, the nonparametric Wilcoxon-Mann-Whitney test and hierarchical cluster analysis were used for choosing the significant miRNAs. Power analysis was used to calculate the number of cases in the training and validation cohort. For the data obtained by qPCR, the nonparametric Mann-Whitney test was used for the comparison between cirrhosis and control. Kruskal-Wallis test was used to compare the expression level among more than two groups. A stepwise logistic regression was used to establish a model as a surrogate marker to diagnose the cirrhosis. Area under the ROC (receiver operating characteristic) curve (AUC) was used as an accuracy index for evaluating the diagnostic performance of the significant miRNAs. The linear regression was used to explore the casual relationship between the expression levels of miRNAs and some clinical indicators. *P*<0.05 was considered statistically significant.

## Results

### Plasma miRNA Profiling and Data Analysis

**Figure 2:**
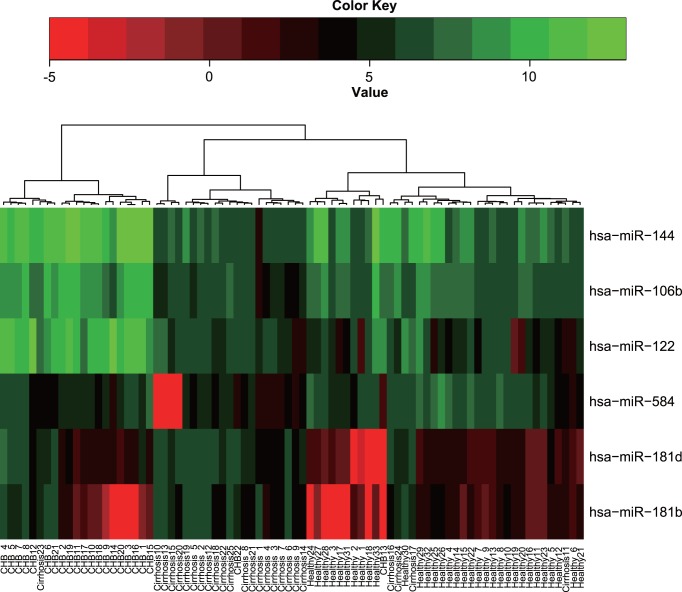
Hierarchical cluster analyses of the significantly regulated six miRNAs. Heat map colors represent the relative miRNA expression as indicated in the color key. bright green: over-expression; black: no change; bright red: under-expression.

The expression profiles of the human miRNAs were determined by a microarray containing probes for 723 human miRNAs between the CHB-related cirrhosis and control groups. Of the analysis, Mann-Whitney test was used to select differentially expressed miRNAs in the pairwise comparison of CHB-related cirrhosis and CHB groups, as well as CHB-related cirrhosis and healthy groups, respectively. There were three miRNAs, including miR-106b, miR-122, and miR-144, with significantly lower expression in CHB-related cirrhosis group than in the CHB group (fold change = 0.06–0.11; *P*<10^−8^). By contrast, miR-181d and miR-181b was significantly up-regulated (fold change = 13.0–14.4; *P*<1.5×10^−6^) and miR-584 was down-regulated (fold change = 0.1; *P*<1.5×10^−6^) in CHB-related cirrhosis group compared with the healthy group. Thus, a list of six differentially produced miRNAs was constructed as candidates for further investigation via qPCR (Table S3 in File S1). Hierarchical clustering analysis was used to check out the grouping potency of the six significant miRNAs (Fig. 2).

### Differential Expression Profile of 6 Selected microRNAs

The six candidate miRNAs selected from the microarray analysis were primary confirmed with qPCR in an independent cohort of 41 plasma samples. Three out of them passed the quality control, but only miR-106b and miR-181b had significantly different expression between the CHB-related cirrhosis and control groups (Table S4 in File S1). The expression profile of the two miRNAs was further confirmed in 141 additional plasma samples calculated by power analysis.

### MicroRNA Expression Profile in the Training Set

**Figure 3:**
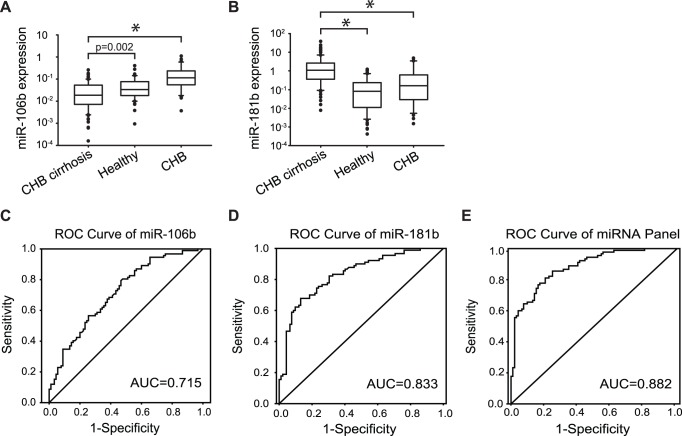
Plasma miR-106b and miR-181b levels are altered significantly in patients with CHB cirrhosis in the training phase. Plasma miR-106b (A) and miR-181b (B) levels were measured in 182 samples by qPCR. Mann Whitney U test was used to determine statistical significance. Represented in the box plots are median, interquartile range, and the data range. ROC curve analysis showed the diagnostic power of miR-106b (C), miR-181b (D), and the miRNA classifier (E). *P<0.05.

The combined 182 samples were used as the training set for the construction of the miRNA panel for diagnosis of liver cirrhosis. Low expression of miR-106b (*P*<0.001, fold change = 0.354) and high expression of miR-181b (*P*<0.001, fold change = 8.276) were observed in patients with CHB-related cirrhosis compared with those in the control group (Fig. 3A and B, Table S5 in File S1). Receiver operating characteristic (ROC) curves were constructed to obtain the relation between the sensitivity and specificity of these two miRNAs at varying cutoff levels. The AUC for miR-106b and -181b were 0.715 (95% CI, 0.641–0.789; sensitivity = 0.804, specificity = 0.522) and 0.833 (95% CI, 0.775–0.892; sensitivity = 0.678, specificity = 0.870), respectively (Fig. 3C and D).

To further evaluate the diagnostic value of the two miRNAs, a stepwise logistic regression analysis (with forward LR method) was applied. Both miR-106b and miR-181b turned out to be the significant predictors, and the risk score of being diagnosed with CHB-related cirrhosis from the logit model based on these two miRNAs. It could be a potential classifier for detecting liver cirrhosis, and the formula of the classifier is as follow: Risk score = 1.763 × miR181b - 14.622× miR106b - 0.367. The ROC curve of the classifier has an AUC of 0.882 (95% CI, 0.834, 0.929; sensitivity = 0.856, specificity = 0.750; Fig. 3E).

### Validating the miRNA Classifier

**Figure 4:**
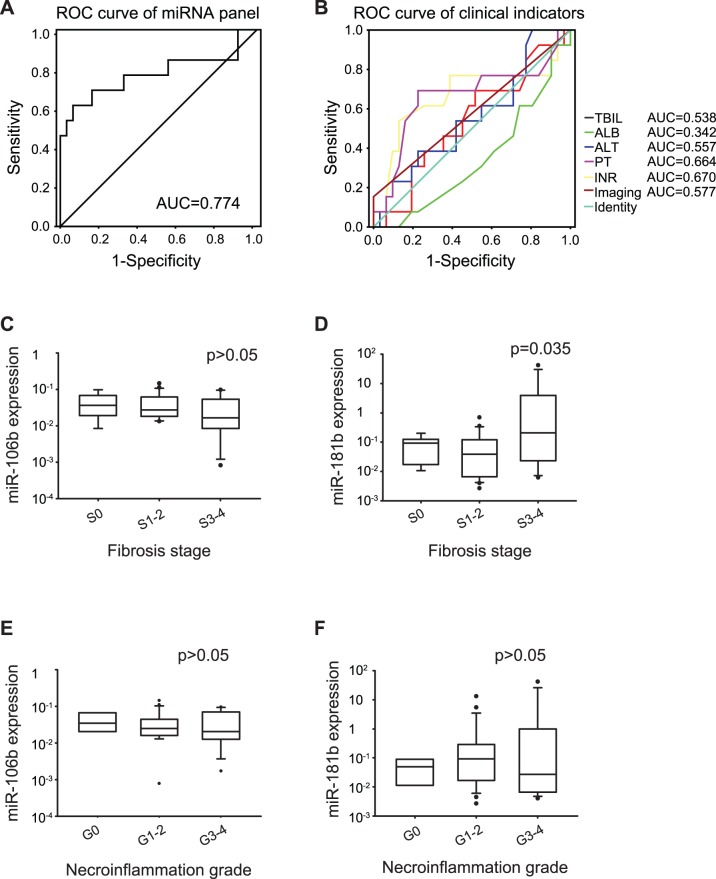
Plasma miR-106b and miR-181b levels in CHB-related silent cirrhosis cohort. ROC curve analysis showed that the diagnostic power of miRNA classifier (A) was higher than the diagnostic powers of single clinical indicators (B). Kruskal-Wallis test was used to determine the statistical significance of miR-106b (D and E) and miR-181b (C and F) in different fibrosis and necroinflammation grades. TBIL, total bilirubin; ALB, albumin; ALT, alanine aminotransferase; PT, prothrombin time; INR, international normalized ratio.

The classifier constructed in the training phase was next used to predict the probability of being diagnosed with CHB-related silent cirrhosis for an independent validation data set (44 samples). In this study CHB-related silent cirrhosis (i.e. exclusive of decompensated liver cirrhosis) was defined as no clinical signs or symptoms plus no biochemical evidence of cirrhosis, and according to the Scheuer scoring system for staging liver fibrosis (S0, S1, S2, S3 and S4) [15], [16], it presents at stage S3–4 (severe fibrosis to cirrhosis). At this stage liver biopsy specimens were obtained from all participants to identify their stage of development. Similarly, the predicted probability was used to construct the ROC curve. The AUC of the classifier was 0.774 (95% CI, 0.589–0.959; sensitivity = 0.615, specificity = 0.935; Fig. 4A), much higher than the AUC of single clinical indicators (including total bilirubin, albumin, alanine aminotransferase, prothrombin time, international normalized ratio and imaging) (Fig. 4B; Table S6 in File S1), indicating the improved diagnostic performance of the classifier compared with clinical indicators.

To investigate whether there is a relation between the classifier and liver necroinflammation or fibrosis, we compared the relative expression of miR-106b and miR-181b in different grades of necorinflammation and fibrosis. A statistically significant difference of miR-181b levels was observed in patients with different stages of fibrosis (*P* = 0.035; Fig. 4C). However, no statistically significant difference of the miR-106b levels was found in patients with different stages of fibrosis (*P*>0.05, Fig. 4D); even though its concentration tended to be lower in patients with severe fibrosis and silent cirrhosis. Further, no significant difference of miR-106b and -181b levels was observed in patients with different grades of necorinflammation (*P*>0.05; Fig. 4, E and F).

### Validating the miRNA Classifier in Non-CHB Cirrhosis Patients Cohort

**Figure 5:**
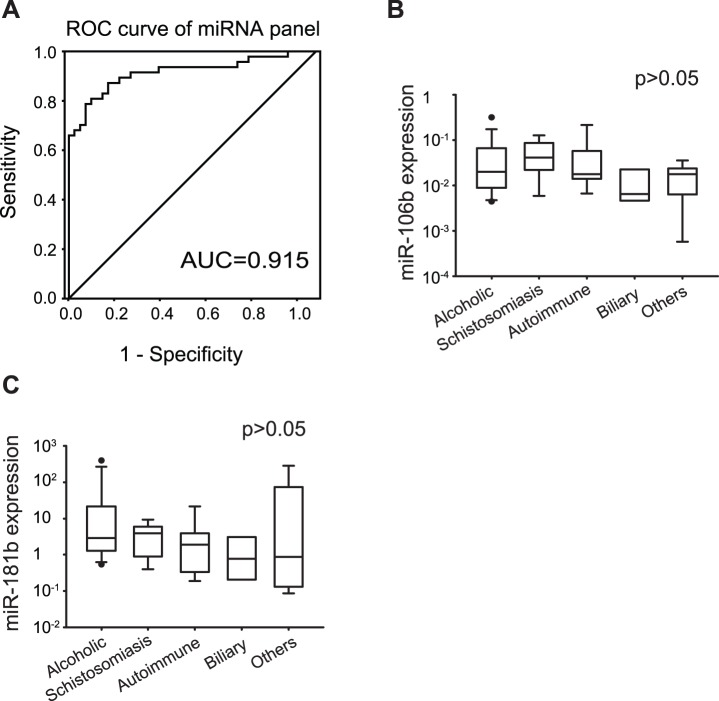
Plasma miR-106b and miR-181b levels in non−CHB-related cirrhosis cohort. ROC curve analysis showed that the diagnostic power of the miRNA classifier was significant high in non-CHB cirrhosis cohort (A). There was no statistic difference between the two miRNAs levels in patients with different types of cirrhosis (B and C), indicating that the dysregulated levels of plasma miRNAs were not etiology-specific.

To investigate whether it was suitable for all kinds of liver cirrhosis, the diagnostic performance of the classifier was validated in a cohort containing non-CHB-related cirrhosis patients, non−CHB-related chronic liver disease patients, and healthy controls. The predicted AUC of the logit model was 0.915 (95% CI, 0.854–0.976; sensitivity = 0.787, specificity = 0.932; Fig. 5A). No statistic difference was observed in the levels of the two miRNAs in patients with different kinds of cirrhosis, indicating that dysregulated levels of miRNAs may irrelevant to the etiology (*P*>0.05; Fig. 5, B and C).

### Liner Regression between miRNAs Level and Clinical Indicators

A liner regression was also used to determine whether there is an association between miRNAs level and clinical indicators, including total bilirubin, albumin, alanine aminotransferase, prothrombin time, and international normalized ratio. The relative expressions of miRNAs, which were used as a dependent variable, were logarithmic transformed to make the data distributed in a normal fashion. However, no significant causal relationship was observed between miRNAs expressions and all these clinical indicators (Table S7 in File S1).

## Discussion

A large number of miRNAs are proved to be present in circulation. They exhibit different profiles in patients with diverse diseases, including acute myocardial infarction [17], [18], ectopic pregnancy [19], rheumatic diseases [20] and various types of malignancies [11], [21], [22]. All these findings bear a resemblance to circulating miRNAs serving as a reliable noninvasive biomarker for the detection of diseases. In the present study, we confined our attention to the plasma miRNA and expected to establish their contribution to the patients with liver cirrhosis, which leads to a poor outcome and end-stage liver disease [1].

Studies have shown that miRNAs can be expressed in a tissue-specific or cell-specific manner. If the liver injury is transient and acute, the hepatocytic changes may be self-limited and undetectable. However if the injury is sustained or chronic, chronic inflammation and accumulation of extracellular matrix persist, leading to the detectable fibrosis-related miRNA in circulation. Therefore, our biomarker discovery strategy with an expectation to reduce the need for biopsy is divided into three phases: (1) Discovery phase: to identify candidate markers through microarray profiling of miRNAs in CHB-related cirrhosis, CHB and healthy individuals. (2) Training phase: to test 6 candidate miRNAs selected from global screening, and then, to further refine 2 selected miRNAs differential expressed in the three groups. (3) Validation phase: to validate the miRNA biomarkers in two independent cohorts all with biopsy containing CHB-related silent cirrhosis and controls, as well as non−CHB-related cirrhosis and controls. Herein, our findings showed that the combined miR-106b and miR-181b have a satisfactory efficiency in detecting patients with CHB-related cirrhosis, particularly those at early stage. In addition, our results also verified that the two miRNAs are not etiology-specific.

Recent studies have determined that the potential use of circulating miRNAs in the diagnosis of liver cirrhosis [23]–[26]. Functional studies of miRNAs in liver tissue may be helpful for evaluating miRNAs as indicators of cirrhosis. Recent research have showed that TGF-β1, a prominent pro-fibrogenic cytokine [27], can induce the expression of miR-181b in hepatic stellate cell. Additionally miR-181b can promote hepatic stellate cells proliferation by targeting p27 [26]. Clinical study on this miRNA in liver cirrhosis, however, are current unavailable. miR-106b represented another regulated miRNA in patients with chronic liver diseases. Although limited information is available regarding miR-106b in liver cirrhosis, its molecular mechanisms and roles were most studied in other diseases. It has been found that miR-106b negatively regulates a lot of gene expression, such as TGF-β, p21/CDKN1A and ULK1, at post-transcriptional level [28]–[31]. Among these targets, TGF-β could induce the expression of connective tissue growth factor (CTGF), which in turn enhance the signaling of TGF-β, along with a lot of other pro-fibrotic factors [32]. Further, as key modulators of TGF-β signaling, the miR-106b-25 cluster and its paralog–miR-17−92 cluster–have been proved their physiologic and pathophysiologic role in the control of liver apoptosis [33]. miR-18a encoded by miR-17−92 cluster family has also been proved to be an anti-fibrotic miRNA in various organs, including heart, lung and liver, by targeting CTGF and other factors [30], [34]. The implicit assumption underlying both studies mentioned above is that miR-106b may be participated in the development of liver cirrhosis. In our study we found that plasma miR-106b level significantly reduced in patients with either CHB-related or non−CHB-related cirrhosis, with no causal relationship with clinical indicators of liver function. These results indicated that aberrant expression of miRNA-106b is more likely associated with fibrogenesis rather than the cause of hepatocyte injury. Thus future researches are necessary to identify the targets of miR-181b and miR-106b and the related mechanism that regulates miRNA expression in liver fibrogenesis.

Although the miRNA classifier had a high diagnostic value in our research, there are still some limitations. As the difficulty in sample collection, the number of patients with liver biopsy prevented us from testing in a more detailed classification. Further, we validated the classifier in a relative small non−CHB-related cirrhosis cohort to demonstrate its role as etiology-independent biomarkers, thus we still need to enlarge sample size of each kind of non−CHB-related cirrhosis at different stage for precise evaluation in future.

In summary, we have demonstrated for the first time that the profile of 2 plasma miRNAs, miR-181b and miR-106b, has potential to serve as a noninvasive biomarker for diagnosing liver cirrhosis, particularly those at early stage. More importantly, data from ours, in which it is possible to differentiate between patients with silent and decompensate cirrhosis, may help guide the clinician when determining what types of triage should be used. Aside from these diagnostic examples of how miRNA expression patterns may be able to be used clinically, the ability of miRNAs to affect multiple genes in various pathways make them a logical target for investigation of novel antifibrotic therapies.

## Supporting Information

File S1 **Figure S1,** Process for selection of candidate miRNAs. CHB, chronic hepatitis B; CHB cirrhosis, CHB-related cirrhosis; qPCR, quantitative real-time PCR. **Table S1,** General inclusion criteria. **Table S2,** The primer sequences. **Table S3,** Candidate miRNAs from microarray result. **Table S4,** Evaluation of the expression of 6 selected miRNAs by qPCR. **Table S5,** Differentially expressed miRNA in the training phase. **Table S6,** The diagnose power of single clinical indicators. **Table S7,** Liner regression between miRNAs level and clinical indexes.
(PDF)
