# Supplementary material for: Circulating microRNAs as a Fingerprint for Liver Cirrhosis
Source: PLoS One. 2013 Jun 21;8(6):e66577. doi: 10.1371/journal.pone.0066577 (PMC3689750; doi:10.1371/journal.pone.0066577)
Supplement: File S1 — Figure S1, Process for selection of candidate miRNAs. CHB, chronic hepatitis B; CHB cirrhosis, CHB-related cirrhosis; qPCR, quantitative real-time PCR. Table S1, General inclusion criteria. Table S2, The primer sequences. Table S3, Candidate miRNAs from microarray result. Table S4, Evaluation of the expression of 6 selected miRNAs by qPCR. Table S5, Differentially expressed miRNA in the training phase. Table S6, The diagnose power of single clinical indicators. Table S7, Liner regression between miRNAs level and clinical indexes. (PDF) [file pone.0066577.s001.pdf]

Table S1. General inclusion criteria*

**General inclusion criteria**
1. Age ≥18 years and ≤80 years
2. No severely ill and with a capability to give informed consent
3. No other serious illness such as heart attacks, renal inadequacy, leukemia and cancer
**Healthy donors (healthy group)**
1. Had the medical check-up in Zhongshan Hospital and in healthy condition
**Chronic hepatitis B patients (CHB group)**
1. HBsAg-positive > 6 months
2. Persistent or intermittent elevation in ALT/AST levels
3. Liver biopsy showing chronic hepatitis with moderate or severe necroinflammation without cirrhosis
4. HBeAg positive or HBV DNA positive
**Non-CHB chronic liver disease (non-CHB CLD group)**
1. Persistent or intermittent elevation in ALT/AST levels
2. Chronic liver disease symptoms, such as liver palms, spider nevus and hepatosplenomegaly
3. Liver biopsy showing chronic hepatitis with moderate or severe necroinflammation without cirrhosis
4. No HBV infection before
**Cirrhosis patients (cirrhosis group)**
1. Had the liver biopsy and diagnosed by two experienced pathologists
2. Reduced serum albumin/raised ALT and AST, with hypersplenism/gastroesophageal varices/hepatic encephalopathy/ascites
3. At least two image reports (ultrasound B, CT or MRI) can support the diagnosis
*Diagnosis of cirrhosis depends on one of the term above, and can be divided into different subgroups according to the following causes:
1. CHB cirrhosis: with a history of CHB or HBsAg positive
2. Alcoholic liver cirrhosis: a history of alcohol abuse or excess with the laboratory tests exclude other etiologies
3. Schistosomiasis cirrhosis: a history of schistosomiasis with a typical performance in image
4. Autoimmune cirrhosis: total serum globulin or γ globulin or IgG concentrations greater than 1.5 times the upper normal limit, or seropositivity for ANA, SMA, or anti LKM-1 antibodies at titers greater than 1:80. Seronegativity for AMA. Exclusion other etiologies.
5. Primary biliary cirrhsosis: biochemical evidence of cholestasis with elevation of alkaline phosphatase activity; presence of AMA; and histopathologic evidence of nonsuppurative cholangitis and destruction of small or medium-sized bile ducts
6. Others: such as gryptogenic cirrhosis, HCV-related cirrhosis

*see American Association for the Study of Liver Disease Practice Guidelines, 2009; CHB, chronic hepatitis B; HBsAg, hepatitis B surface antigen; ALT, alanine aminotransferase; AST, aspartate aminotransferase; ANA, anti-nuclear antibodu; SMA, smooth muscle antibody; LKM-1, Liver-kidney microsom-1; AMA, anti-mitochondrial antibody.

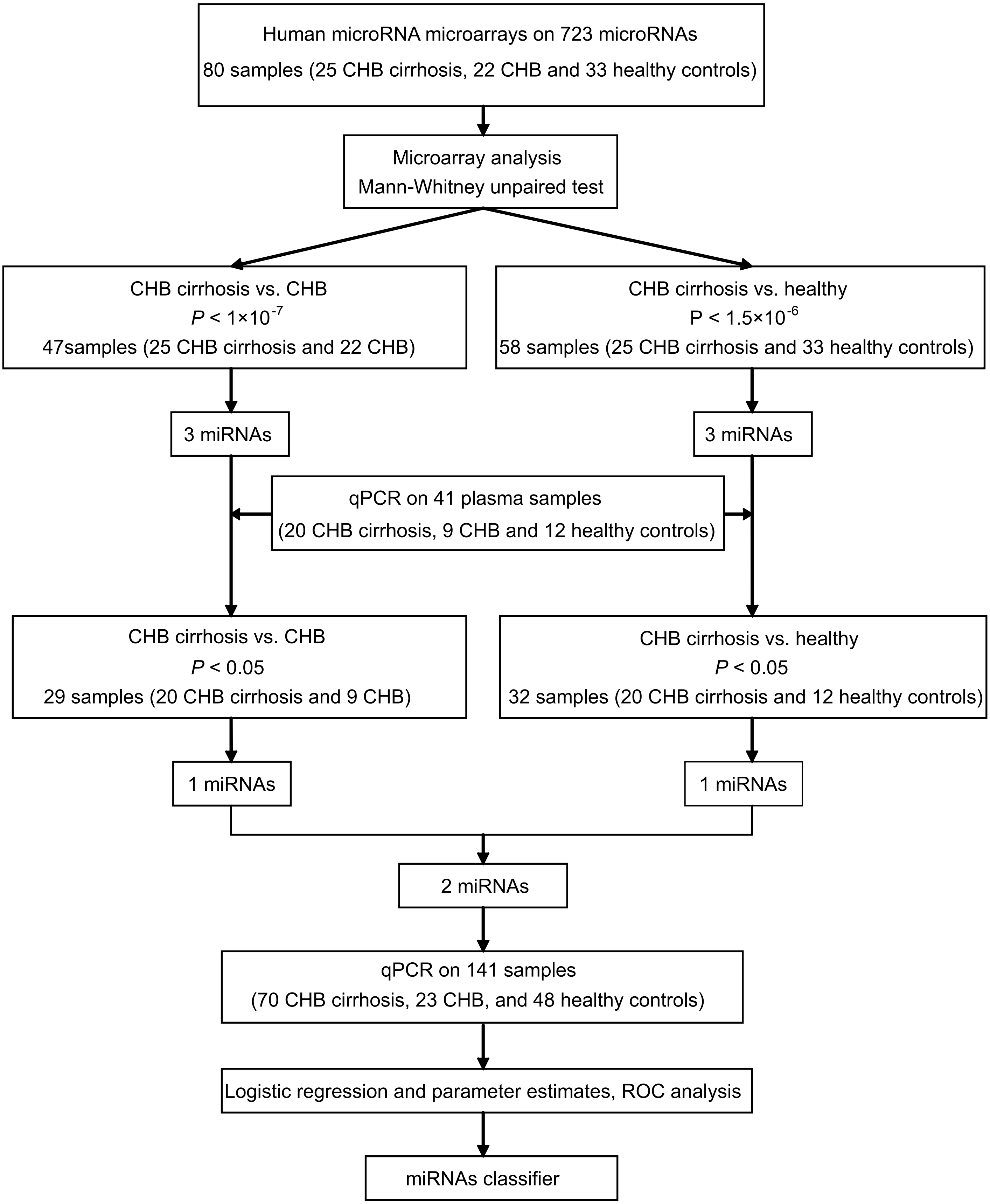

Figure S1

Process for selection of candidate miRNAs. CHB, chronic hepatitis B; CHB cirrhosis, CHB-related cirrhosis; qPCR, quantitative real-time PCR.

Table S2. The primer sequences

Primers	Sequences
miR-16-5p	5'-TAGCAGCACGTAAATATTGG-3'
miR-106b-5p	5'-GTCCAGTGCTGACAGTGCAGATA-3'
miR-122-5p	5'-TCTGGAGTGTGACAATGGTGTTTG-3'
miR-144-3p	5'-CCCAGCCTCCAGTATAGATGATGT-3'
miR-181b	5'-GGCGCGAACATTCATTGCTGTC-3'
miR-181d	5'-CCCGCGAACATTCATTGTTGTC-3'
miR-584-5p	5'-TTATGGTTTGCCTGGGACTGAG-3'

Table S3. Candidate miRNAs from microarray result

	Cirrhosis vs CHB			Cirrhosis vs healthy		
	***P* value**	**Fold change**	**regulation**	***P* value**	**Fold change**	**regulation**
**miR-106b**	5.05E-8	0.108	down	4.26E-5	0.336	down
**miR-122**	1.13E-8	0.057	down	0.061595526	1.8006091	up
**miR-144**	7.85E-8	0.085	down	0.026537156	1.9502187	down
**miR-181b**	0.002218768	11.088	up	9.07E-9	12.979672	up
**miR-181d**	0.005231245	2.911	up	3.96E-10	14.36861	up
**miR-584**	0.015033541	0.242	down	1.02E-6	0.099	down

Table S4. Evaluation of the expression of 6 selected miRNAs by qPCR

	Cirrhosis vs CHB			Cirrhosis vs healthy			Cirrhosis vs controls*		
	***P* value**	**Fold change**	**regulation**	***P* value**	**Fold change**	**regulation**	***P* value**	**Fold change**	**regulation**
**miR-106b**	0.003	0.161	down	0.022	0.368	down	0.001	0.237	down
**miR-122**	0.024	0.917	down	0.471			0.442		
**miR-144**	0.588			0.293			0.686		
**miR-181b**	0.005	8.61	up	0.002	12.03	up	<0.001	10.977	up
**miR-181d**	0.437			0.907			0.611		
**miR-584**	0.383			0.669			0.442		

*controls including healthy and CHB subjects.

Table S5. Differentially expressed miRNA in the training phase

	Cirrhosis vs CHB			Cirrhosis vs healthy			Cirrhosis vs controls*		
	**regulation**	**Fold change**	***P* value**	**regulation**	**Fold change**	***P* value**	**regulation**	**Fold change**	***P* value**
**miR-106b**	down	0.191	<0.001	down	0.649	0.002	down	0.354	<0.001
**miR-181b**	up	4.47	<0.001	up	15.12	<0.001	up	8.276	<0.001

*controls including healthy individuals and CHB patients

Table S6. The diagnose power of single clinical indicators

	AUC	Asymptotic 95% CI		Specificity	Sensitivity
		Lower Bound	Upper Bound		
**miRNA classify**	0.774	0.589	0.959	0.935	0.618
**TBIL**	0.538	0.352	0.725	0.484	0.692
**ALB**	0.342	0.170	0.515	0.097	0.923
**ALT**	0.557	0.370	0.745	0.194	1.000
**PT**	0.664	0.464	0.863	0.774	0.692
**INR**	0.670	0.467	0.873	0.871	0.538
**Imaging**	0.577	0.380	0.773	1.000	0.154

CI, confidence interval; miRNA, microRNA; TBIL, total bilirubin; ALB, albumin; ALT, alanine aminotransferase; PT, prothrombin time; INR, international normalized ratio.

Table S7. Liner regression between miRNAs level and clinical indexes

**Clinical indexes**	**miR-106b**		**miR-181b**	
	**regression coefficient**	***P* value**	**regression coefficient**	***P* value**
**TBIL**	-0.002	0.230	0.003	0.183
**ALB**	0.007	0.739	-0.036	0.227
**ALT**	-0.002	0.072	-0.002	0.324
**PT**	0.009	0.842	0.027	0.671
**INR**	0.112	0.834	0.335	0.667

TBIL, total bilirubin; ALB, albumin; ALT, alanine aminotransferase; PT, prothrombin time; INR, international normalized ratio.
